# Publication disparities in gender-transformative sexual and reproductive health in West Africa

**DOI:** 10.4102/jphia.v17i1.1717

**Published:** 2026-04-29

**Authors:** Mat Lowe, Kéfilath Bello, Bernice Gyawu, John K. Krugu, Aminatou I. Assoumane, Ngianga B. Kandala, Nathalie Sawadogo

**Affiliations:** 1 African Population and Health Research Center, Dakar, Senegal; 2 Centre for Research in Human Reproduction and Demography, Cotonou, Benin; 3 Alliance for Reproductive Health Rights, Accra, Ghana; 4 Youth Harvest Foundation-Ghana, Accra, Ghana; 5 GRADE Africa, Niamey, Niger; 6 Demographic Dynamics and Population Health Unit, West Africa Regional Office, African Population and Health Research Centre, Ottawa, Ontario, Canada; 7 Institut Supérieur des Sciences de la Population, Université Joseph Ki-Zerbo, Ouagadougou, Burkina Faso

**Keywords:** gender-transformative approaches, health, West Africa, publication, disparities

## Abstract

This opinion paper is informed by a scoping review and validated through a multi-stakeholder workshop. In May 2024, the Gender Transformation for Africa, an African-led research partnership that includes programme implementers and researchers who are working on gender-transformative research projects to advance sexual and reproductive health (SRH) in Africa, convened a 3-day workshop in Cape Town, South Africa. The aim of the workshop was to validate a scoping review conducted by the School of Public Health at the University of the Western Cape. The scoping review aimed to examine patterns of leadership in authorship of gender-transformative SRH research and programming across Africa and focused on authorship, funding and geographic context. This review revealed significant regional disparities in the publication of gender-transformative SRH literature across Africa. Compared with the Southern and Eastern Africa regions, the West Africa region has the smallest representation of authorship, with only five articles, none of which are from Francophone West Africa. Some of the underlying factors behind the disparities in the publication of gender-transformative SRH research in West Africa are discussed in this opinion paper, and ways to address the reported imbalances are suggested. This article draws on the discussions and perspectives shared by the workshop participants, along with insights from the literature and other West African researchers.

## Introduction

Over the past few decades, researchers and development practitioners have made significant efforts to shift health and development interventions from being gender-neutral to gender-sensitive and gender-transformative.^1^ Gupta provides a more familiar definition of these concepts. While gender-neutral interventions are those that do not take gender into consideration, Gupta^2^ defined gender-sensitive interventions as those that recognise the differing needs and constraints of women and men. Gender-transformative interventions, in contrast, are those interventions that seek to reshape gender relations to be more gender equitable through gender-transformative approaches that challenge the unequal structures and norms that perpetuate disparities. Despite its political contestation, especially in cross-cultural contexts, the application of gender-transformative approaches in health and development interventions has increased exponentially over the last three decades.^3^ However, disparities in who produces knowledge on gender-transformative approaches persist.

In May 2024, the Gender Transformation for Africa (GT4Africa), an African-led research partnership based at the University of the Western Cape, convened a 3-day feminist fiesta workshop in Cape Town, South Africa. The workshop included 15 programme implementers and researchers from the GT4Africa who are working on gender-transformative research projects to advance sexual and reproductive health (SRH) in Africa and funded by the International Development Research Centre (IDRC). The workshop aimed to validate a scoping review conducted by the School of Public Health at the University of the Western Cape in South Africa. The scoping review aimed to examine imbalances in authorship, geographic and institutional contexts and funding sources in research on gender approaches to SRH in Africa.^4^ The review included 45 publications from 2012 to 2022 in PubMed and Scopus, along with consultations with African gender and health experts. Publications were relevant and included in the review if they employed gender approaches in SRH research and programming in Africa. The reviewed articles were situated in Southern Africa, Eastern Africa, Western Africa and Northern Africa. The findings revealed significant regional disparities in the publication of gender-transformative SRH research across Africa. It showed that South African authors account for 21.2% of first authorship and 28.0% of last authorship, which is more than twice the share of authors from other African regions, notably Northern and Western Africa. Compared with the Southern and Eastern Africa regions, the West Africa region has the lowest representation, accounting for 11.1% of first and second authorship, with only five articles, none of which are from Francophone West Africa. Potential factors that may explain the disparities in the publication of gender-transformative SRH research in West Africa are discussed in this opinion paper, and recommendations for addressing these disparities are provided. The objective of this article is to contribute to critical discourse on disparities in the publication of gender-transformative SRH research in West Africa. The article draws on the discussions and perspectives shared by the participants in the feminist fiesta workshop, along with insights from the literature and other West African researchers.

## Potential factors that may explain the disparities in the publication of gender-transformative sexual and reproductive health in West Africa

The workshop participants attributed the publication imbalances in gender-transformative SRH research in West Africa to a diverse set of intertwined factors operating at the global, regional, national and institutional levels. These include: (1) colonial legacies, (2) the biomedical dominance of national health research, (3) backlash and (4) legal and social constraints, and vulnerability against research on gender equality and SRH. We discussed each of these factors in detail.

### Colonial legacies

Workshop participants noted that the enduring presence of colonial legacies, which sustain the dominance of the English language in global academic publishing, is a major factor contributing to the publication imbalance in gender-transformative SRH in West Africa. As West Africa is predominantly French-speaking, participants noted that this factor presents a significant challenge for Francophone West African researchers who wish to undertake gender-transformative SRH research and publish their work in English. This perspective is consistent with a previous commentary article. The authors of this article argued that anglophone donors and international nongovernmental organisations that have historically implemented SRH programmes perpetuate English dominance in the SRH field.^5^ Francophone countries make up a substantial share of nations receiving SRH donor funding; yet French speakers remain linguistically isolated.^5^ This is due to language barrier and the scarcity of global and public health journals where they can publish their work in French, as explained by two researchers. The researchers argued that the environment of scientific journals in French-speaking West African countries is such that local academic journals, due to language and limited resources, are not all online, or, when they are, their articles are not updated in real time. As a result, online systematic reviews miss some French-language articles. The limited visibility of local French journals in West Africa is not due to a lack of research but to an indexation gap that constitutes systematic exclusion. A survey of sixty-three (*n* = 63) African medical journals, for instance, found that in French-speaking Africa, fewer than 10 medical journals publish regularly, and only five are indexed in Medline.^6^ This factor may constitute a major barrier to the visibility of gender-transformative SRH research published in French. Consistent with this perspective, previous studies have underscored the dominant influence of the English language in academic knowledge production and translation as problematic. For example, a bibliographic analysis of publications from low- and middle-income countries (LMICs) in nine prominent medical and global health journals during 2014–2016 found that 26.2% of the 416 Africa-based publications were from the East Africa sub-region.^7^ The authors attributed this disparity to the legacy of the English language and the relative competitive advantage built up over time by researchers in this region. Another article exploring language inequities in scholarly publishing highlights the dominance of English, emphasising the disparities this creates for non-English-speaking researchers and the consequent impact on the diversity and inclusivity of scientific discourse.^8^

### The biomedical dominance of national health research

The concentration of funding in biomedical research can influence national research agendas, institutional incentives and publication outputs, resulting in gender-transformative SRH research under-resourced. For instance, since 2002, the Global Fund has invested US$6.9 billion in Western and Central Africa targeting three diseases (malaria, tuberculosis [TB] and human immunodeficiency virus [HIV] and/or acquired immunodeficiency syndrome [AIDS]).^9^ This amount constitutes 18% of the total Global Fund investments, and approximately 53% of these investments were directed to malaria, including malaria research.^9^ This is due to malaria’s 40% contribution to the global disease burden, particularly among children in West Africa.^9^ In addition, workshop participants argued that while funding gender and health research may be a priority, it is not a ‘top research and political priority’ for West African governments and for most donors compared with biomedical research. This perspective from participants reflects the situation of research funding and partnership in Africa, where North–South partnerships are most prevalent in biomedical research.^10^ This dominance of biomedical research may limit consideration of gender and other social determinants of SRH research by governments. It may also mean that while funders such as the Global Fund can integrate gender-transformative work into their supported biomedical programmes, such as Malaria and HIV research, this work is rarely published as the primary focus or considered ‘publishable science’ by major health journals.

The workshop participants also noted that in addition to the biomedical dominance of national health research, the funding infrastructure in West Africa (including the lack of local investment and reliance on external support) is a major problem. They argued that West African governments rely heavily on external donors for support, and these donors may not prioritise gender-transformative research over other issues they perceive as more significant, such as biomedical research. This perspective is consistent with the finding that a larger proportion of research funding in West Africa goes to biomedical research, including malaria, TB and HIV.^9^

### Backlash

In most contexts across West Africa, traditional religious and conservative norms and values are deeply rooted. Given this social dynamic, the workshop participants argued that most West African researchers may view gender-transformative research as a no-go area, as exemplified by Ludovic Lado in ‘Politics of Gender Reform in West Africa: family, religion and the state’.^11^ Lado opines that attempts to implement gender reforms in West Africa can be met with suspicion because the people perceive such reforms as a Western imposition and imperialism that could subvert traditional cultural structures of authority, community and family. The participants noted that in West Africa, the term ‘gender’ is frequently linked to promoting the agenda of the community of people with diverse sexual orientations and gender identities. This often incites strong, sometimes violent opposition from community and religious groups who clearly lash back at ideas of gender equality by presenting these as corrupt and Western imperialism.^12^ This misinterpretation may deter local and regional funders from supporting gender-related health research or publications because of concerns about controversy or social backlash. Consequently, this limits the potential impact of such research and publications and could increase backlash against gender-transformative research that seeks to challenge and transform the underlying power structures and societal norms perpetuating gender inequality.^13^ Backlash, in this sense, is a predictable expression of the defence of established and unjust patterns of gender that calls for the deconstruction and decolonisation of power structures, including donor conditionalities and epistemic exclusion.^14,15^

### Legal and social constraints, and vulnerability against research on gender equality

The workshop participants identified factors that were more inward-looking, highlighting local restrictions and associated vulnerabilities. These include tensions between the transformative aspirations of researchers in gender equality and SRH and the patriarchal and conservative contextual realities across many West African countries, especially regarding legal and policy frameworks and sociocultural and religious norms. Participants reiterated the daunting nature of conducting research in this environment, particularly given the threat of security risks posed by unrest or terrorism in certain parts of the continent, and reported using alternative, less contentious language suited to their context to discuss sensitive issues (e.g. condom use, safe sex and sexuality education). One of the workshop participants explained that, in a project he was leading on strengthening access to quality comprehensive sexuality education (CSE) among adolescents, the project team had to drop the phrase ‘comprehensive sexuality education’ from the project title and replace it with ‘comprehensive health education’ to reach the gender-transformative goals of the project while avoiding potential social backlash. Another participant said that they had to replace in their project title the term ‘child marriage’ with ‘early marriage’ when referring to marriage below the age of 18 to make their project acceptable to the communities and political decision-makers. These situations demonstrate the culturally sensitive nature of research on gender and gender equality issues in West Africa and can help explain the relatively low number of articles from West Africa included in the scoping review.^4^

But other factors could also explain the disparities in the publication of gender-transformative SRH research in West Africa. These include the power dynamics within collaborative research partnerships, in which the Northern partners assume the roles of donors and conceptualisers, steering the research direction, while the Southern partners occupy a peripheral position as local implementers.^16^ Additionally, African scholars frequently face marginalisation within global knowledge systems, which tend to prioritise outputs from high-income countries, and these disparities are further shaped by entrenched inequalities in research infrastructure (RI), funding and visibility.^17^ Together, these factors could create an environment in which gender-transformative SRH research and publication face significant challenges in West Africa. The consequences of these disparities are far-reaching, necessitating deliberate strategies for change. Workshop participants suggested various strategies to address the disparities in the publication of gender-transformative SRH in West Africa.

## Addressing the disparities in the publication of gender-transformative sexual and reproductive health in West Africa

To address the disparities in the publication of gender-transformative SRH literature in West Africa, the workshop participants suggested providing capacity-building training for West African researchers in gender and health research. Existing research capacity strengthening programmes, such as the Consortium for Advanced Research Training in Africa (CARTA) and the African Research Initiative for Scientific Excellence (ARISE), have demonstrated the effectiveness of regional training programmes. The CARTA, a consortium of eight African partner universities, four research institutions and eight non-African partner institutions, which are jointly led by the African Population and Health Research Center (APHRC), has contributed to rebuilding and strengthening the capacity of African researchers and universities to produce world-class researchers, research leaders and scholars in various disciplines.^18^ African Research Initiative for Scientific Excellence has enabled experienced young science, technology, engineering and mathematics professionals to become future leaders in the development, management and operation of advanced RIs, accelerating the development and availability of technological innovation in life sciences.^19^ The workshop participants argued that these training models could be used to strengthen research capacity in gender-transformative SRH research and increase publication outputs on gender-transformative SRH in West Africa. Additionally, because training programmes are more prevalent in Anglophone West Africa than in Francophone West Africa, the workshop participants suggested increasing gender-oriented and bilingual training programmes. They suggested creating a Bilingual Africa Community of Practice and a Community of Practice for the Teaching and Learning of Gender. The participants emphasised that such capacity-building programmes should focus more on supporting Francophone West African researchers, who are often hindered by their limited ability to write and publish in English. Such capacity-building efforts should also be broad enough to include fundraising for research purposes. Additionally, the participants emphasised the importance of fostering a culture of gender and health research and publication among West African researchers. This effort, they argued, should include engaging faith and traditional leaders to mitigate risks to researchers and pushbacks against research on gender equality and SRH. The workshop participants also suggested challenging the dominance of biomedical orientation and promoting the integration of a gender lens in national health research agendas across Africa. Furthermore, they suggested utilising South–South cooperation as a strategy to build capacity and foster research collaboration on SRH through enhanced networking, innovative approaches while supporting the development and sustainability of local and regional knowledge platforms across Africa.

## Reflexive statement

In this article, we have made an important contribution to debates on knowledge production and epistemic injustice in academic global health.^20^ However, it is important to acknowledge that our analysis focuses only on the structural trends driving the imbalance in publications and does not imply a uniform reality across all West African countries. We recognise the diversity within West Africa, including differences in political systems, religious contexts and research capacities.

In addition, we are aware of the positionality of workshop participants and our own perspectives as programme implementers and researchers working on gender-transformative approaches to advancing SRH in West Africa. We are also predominantly Anglophone. Only two of us are bilingual and can speak French and English fluently, which could introduce bias into our analysis.
